# Ivermectin and gemcitabine combination treatment induces apoptosis of pancreatic cancer cells *via* mitochondrial dysfunction

**DOI:** 10.3389/fphar.2022.934746

**Published:** 2022-08-26

**Authors:** Da Eun Lee, Hyeon Woong Kang, So Yi Kim, Myeong Jin Kim, Jae Woong Jeong, Woosol Chris Hong, Sungsoon Fang, Hyung Sun Kim, Yun Sun Lee, Hyo Jung Kim, Joon Seong Park

**Affiliations:** ^1^ Department of Surgery, Gangnam Severance Hospital, Yonsei University College of Medicine, Seoul, South Korea; ^2^ Department of Medical Science, Graduate School of Medical Science, Brain Korea 21 Project, Yonsei University College of Medicine, Seoul, South Korea; ^3^ Department of Medicine, Yonsei University College of Medicine, Seoul, South Korea

**Keywords:** pancreatic cancer, gemcitabine, ivermectin, apoptosis, reactive oxygen species, mitochondrial dysfunction

## Abstract

Pancreatic cancer is an aggressive cancer characterized by high mortality and poor prognosis, with a survival rate of less than 5 years in advanced stages. Ivermectin, an antiparasitic drug, exerts antitumor effects in various cancer types. This is the first study to evaluate the anticancer effects of the combination of ivermectin and gemcitabine in pancreatic cancer. We found that the ivermectin–gemcitabine combination treatment suppressed pancreatic cancer more effectively than gemcitabine alone treatment. The ivermectin–gemcitabine combination inhibited cell proliferation *via* G1 arrest of the cell cycle, as evidenced by the downregulation of cyclin D1 expression and the mammalian target of rapamycin (mTOR)/signal transducer and activator of transcription 3 (STAT-3) signaling pathway. Ivermectin–gemcitabine increased cell apoptosis by inducing mitochondrial dysfunction *via* the overproduction of reactive oxygen species and decreased the mitochondrial membrane potential. This combination treatment also decreased the oxygen consumption rate and inhibited mitophagy, which is important for cancer cell death. Moreover, *in vivo* experiments confirmed that the ivermectin–gemcitabine group had significantly suppressed tumor growth compared to the gemcitabine alone group. These results indicate that ivermectin exerts synergistic effects with gemcitabine, preventing pancreatic cancer progression, and could be a potential antitumor drug for the treatment of pancreatic cancer.

## Introduction

Pancreatic cancer is the fourth leading cause of cancer-related deaths in the world. In addition, the 5-year survival rate is less than 10% (Jia et al., 2019; Hyatt and Powers, 2021; Lee et al., 2021) owing to late diagnosis, frequent metastases, and limited treatment options (Liang et al., 2017). Currently, gemcitabine is one of the standard chemotherapeutic drugs used for treating patients with pancreatic cancer. However, its contribution to increasing the overall survival is negligible due to its low efficacy (Rawla et al., 2019). Therefore, it is necessary to discover novel chemotherapeutic agents and develop effective therapeutic strategies to enhance the tumor susceptibility of gemcitabine to reduce the tumor growth in pancreatic cancer.

Mitochondria play an important role in tumorigenesis by regulating ATP production and apoptosis (Masoud et al., 2020; Fu et al., 2021). Reactive oxygen species (ROS) generation occurs in the mitochondria of cancer cells to support tumor initiation *via* oncogenic changes, such as abnormal cell proliferation, metastasis, and angiogenesis, to avoid apoptosis and overcome hypoxia (Muz et al., 2015). However, excessive accumulation of ROS induces oxidative damage in mitochondria, reduces the mitochondrial membrane potential (MMP), and leads to mitochondrial dysfunction (Aggarwal et al., 2019). Mitochondrial dysfunction promotes apoptosis *via* the release of cytochrome c, which activates the caspase cascade (Lin et al., 2012; Wallace, 2012). In addition, mitochondrial dysfunction induces the excessive production of ROS and bioenergetic failure (Bhatti et al., 2017; Millichap et al., 2021). Thus, cells eliminate dysfunctional mitochondria *via* mitophagy to maintain cellular fitness (Ponraj et al., 2018).

Ivermectin is a U.S. Food and Drug Administration (FDA)-approved antiparasitic drug that is widely used in clinical practice (Zhang et al., 2015). Ivermectin is as a potential anticancer agent against colon cancer, breast cancer, ovarian cancer, melanoma, and leukemia (Wang et al., 2016). It reverses multidrug resistance, inhibits angiogenesis, and decreases mitochondrial biogenesis (Juarez et al., 2018). It also increases ROS generation to induce apoptosis in esophageal squamous cell carcinoma (Xu et al., 2021). Moreover, ivermectin inhibits the serine/threonine kinase (AKT)/mammalian target of rapamycin (mTOR) signaling pathway in breast cancer (Wang et al., 2016). However, the effect of ivermectin on pancreatic cancer and its underlying mechanism are not well understood.

In this study, we investigated the antitumor effects of ivermectin in pancreatic cancer. Interestingly, we found that co-administration of ivermectin and gemcitabine had a significantly more suppressive effect than gemcitabine alone on pancreatic cancer. We also confirmed that the ivermectin–gemcitabine combination induced apoptosis *via* mitochondrial dysfunction and inhibited mitophagy. Moreover, the ivermectin–gemcitabine combination effectively inhibited tumor growth *in vivo*, similar to the gemcitabine alone group. Taken together, the present study suggests that ivermectin, in combination with gemcitabine, could be a promising therapeutic candidate for patients with pancreatic cancer.

## Materials and methods

### Ethics statement

Experiments involving human participants were reviewed by the ethics committee of Gangnam Severance Hospital. All patients provided written informed consent to participate in the study. The animal study protocol was reviewed and approved by the Gangnam Severance Hospital of Yonsei University.

### Cell culture and treatment

Human pancreatic cancer cell lines (MIA PaCa-2 and PANC-1) were purchased from the American Type Culture Collection (ATCC, MD, VA, United States). MIA PaCa-2 and PANC-1 cells were cultured in Dulbecco’s modified Eagle’s medium (DMEM) supplemented with 10% fetal bovine serum (Biowest, MO, United States) and 1% antibiotic-antimycotic reagent (Gibco, MA, United States) at 37°C and 5% CO_2_. Cells were treated with different concentrations of gemcitabine (Yuhan, Seoul, Korea) and ivermectin (Selleckchem, PA, United States).

### Cell viability assay

The cells were seeded in a 96-well plate at a density of 3 × 10^3^ per well and incubated for 24 h. Then, the cells were treated with the indicated concentrations of gemcitabine and ivermectin for 72 h. The growth medium was replaced with 10% water-soluble tetrazolium (WST)-1 reagent (DoGenBio, Seoul, Korea) and incubated for 1 h at 37°C in the dark. The absorbance of each well was measured at 450 nm wavelength using a VersaMax microplate reader (Molecular Devices, CA, United States).

### Patient-derived organoids

Pancreatic tissues were obtained from patients diagnosed with pancreatic cancer at the Gangnam Severance Hospital from 2018 to 2019. Written informed consent was obtained from all patients, and this study was approved by the Institutional Review Board (3-2018-0241). The tissues were chopped and washed with advanced DMEM/F12 (Gibco) supplemented with 1% penicillin-streptomycin (Welgene, Gyeongsan, Korea) and then enzymatically digested with advanced DMEM/F12 supplemented with 0.125 mg/ml dispase II (Wako, VA, United States), 0.1 mg/ml DNase I (Millipore, MA, United States), 0.125 mg/ml collagenase II (Gibco), and 1% penicillin-streptomycin for 1 h at 37°C with shaking (150 rpm). After digestion, the supernatant was filtered through a 70-µm cell strainer (SPL, Gyeonggi-do, Korea) and pelleted *via* centrifugation at 200 × g for 5 min. The pellet was resuspended and mixed with Matrigel (Corning, NY, United States) at a ratio of 1:1 and incubated at 37°C for 10 min to polymerize the matrices. Ivermectin at 4 and 8 μM concentrations was used to treat the cells for 72 h.

### Cell cycle analysis

MIA PaCa-2 cells were seeded in a 6-well-plate at a density of 3 × 10^5^ per well and incubated for 24 h. Then, cells were treated with ivermectin and gemcitabine for 48 h. Treated cells were harvested, fixed in 70% ethanol, and stained with propidium iodide (PI; Sigma-Aldrich, MO, United States) and RNase A (Sigma-Aldrich) for 30 min in the dark. The fluorescence intensity was measured using an FACScanto II flow cytometer (BD Biosciences, NJ, United States). A minimum of 10,000 events were collected on each sample. Cell cycle analysis of DNA histograms was performed with FCS Express Flow Cytometry Software.

### Apoptosis analysis

MIA PaCa-2 cells were seeded in a 6-well-plate at a density of 3 × 10^5^ per well and incubated for 24 h. Then, cells were treated with the indicated concentrations of gemcitabine and ivermectin for 48 h, and stained with the fluorescein isothiocyanate (FITC)-Annexin V Apoptosis Detection Kit I (BD Biosciences), following the manufacturer’s protocols. Briefly, the cells were stained with PI and FITC for 15 min in the dark, and cell apoptosis was analyzed using an FACScanto II flow cytometer (BD Biosciences) and BD FACSDiva Software (version 8.0.3). A minimum of 10,000 events were collected on each sample.

### Reactive oxygen species measurement

MIA PaCa-2 cells were plated on a 6-well-plate at a density of 3 × 10^5^ per well and incubated for 24 h. Then, cells were treated with the indicated concentrations of gemcitabine and ivermectin for 48 h. After treatment, MIA PaCa-2 cells were incubated with 20 μM 2ʹ7ʹ-dichlorodihydrofluorescein diacetate (DCF-DA; Sigma-Aldrich) for 20 min at 37°C in the dark. Cells were washed with PBS twice, and ROS levels were measured by measuring DCF fluorescence using an FACScanto II flow cytometer (BD Biosciences).

### JC-10 staining

MIA PaCa-2 cells were seeded in a 6-well confocal plate at a density of 3×10^5^ per well. MMP was assessed using the Mitochondrial Membrane Potential Kit (Sigma-Aldrich), following the manufacturer’s protocols. Briefly, drug-treated cells were stained with JC-10 dye for 30 min at 37°C in the dark. Buffer B was added, and the cells were then visualized with Confocal Laser Scanning Microscope (Zeiss LSM 780) and ImageJ software.

### Oxygen consumption rate measurement

The Oxygen consumption rate (OCR) was measured using a Seahorse XF24 extracellular flux analyzer (Seahorse Bioscience, MA, United States). MIA PaCa-2 cells were seeded into XF-24 plates at a density of 3 × 10^5^ per well for 24 h and treated with ivermectin and gemcitabine. Then, the cells were incubated XF assay media for 1 h at 37°C in a non-CO_2_ incubator and stressed with sequential addition of 1 µM oligomycin, 2 µM carbonyl cyanide p-(trifluoromethoxy) phenylhydrazone, and a 0.5 µM cocktail of rotenone/antimycin A. The OCR was normalized to total cellular protein concentration.

### Reverse transcription-polymerase chain reaction

MIA PaCa-2 cells were seeded in a 6-well-plate at a density of 3 × 10^5^ per well and incubated for 24 h. Then, cells were treated with the indicated concentrations of gemcitabine and ivermectin for 48 h. RNA was isolated using the TRIZOL reagent (Sigma-Aldrich). Total RNA isolated samples were analyzed *via* RT-PCR using the Maxime RT-PCR premix kit (Intron, Gyeonggi-do, Korea).

### Western blotting analysis

MIA PaCa-2 cells were seeded in a 6-well-plate at a density of 3 × 10^5^ per well and incubated for 24 h. Then, cells were treated with the indicated concentrations of gemcitabine and ivermectin for 48 h. Then, treated cells were lysed using the radioimmunoprecipitation assay buffer. Cell lysates were separated using sodium dodecyl sulfate-polyacrylamide gel electrophoresis and transferred onto polyvinylidene fluoride membranes. After blocking with 5% skim milk for 1 h, the membranes were incubated with the primary antibodies (1:1000) at 4°C overnight, followed by incubation with the horseradish peroxidase (HRP)-conjugated secondary antibodies (1:5000) for 1 h. The protein bands were then exposed to an enhanced chemiluminescent HRP substrate (Thermo Fisher Scientific, United States) and detected on X-ray films. The primary antibodies: Anti-cleaved/pro-caspase 9 (# 56076), anti-cleaved/pro-caspase 3 (# 9662/9661), anti-Bcl2 (# 509), anti-p21 (# 6246), anti-CDK4 (# 601), and anti-CDK6 (# 7961) were purchased from Santa Cruz Biotechnology (Dallas, TX, United States). Anti-Cyclin D1 (# 2922S), anti-PI3Kinase p110 alpha (# 4249T), anti-mTOR (# 2972S), anti-Phospho-mTOR (# 5536S), anti-Phospho-Akt (# 9275S), anti-Akt (# 9272S), anti-Bax (# 2772T), anti-Phospho-STAT3 (# 9145S), anti-STAT3 (# 9139S), were purchased from Cell Signaling Technology (Danvers, MA, United States). Anti-γ-tubulin (#T6557) was purchased from Sigma-Aldrich (St. Louis, MO, United States). HRP-conjugated goat anti-mouse secondary (# 7076S) and HRP-conjugated goat anti-rabbit secondary (# 7074S) antibodies were obtained from Cell Signaling Technology (Danvers, MA, United States).

### Xenograft tumor model

Five-week-old male BALB/c nude mice were purchased from the Model Animal Research Center of Yonsei University (Seoul, Korea). 4 × 10^6^ PANC-1 cells were subcutaneously injected into the left flank of each mouse. When tumors reached approximately 150 mm^3^, mice were randomized into four groups (*n* = 6). Gemcitabine (10 mg/kg) and ivermectin (5 mg/kg) were intraperitoneally injected twice a week for 21 days. Tumor volume was measured using calipers and calculated using the following formula = 0.5 × length × width^2^. On the 21st day, the tumors were harvested, weighed, and fixed in 4% paraformaldehyde. All animal experimental procedures followed the National Institutes of Health Guide for the Care and Use of Laboratory Animals and were performed in accordance with the protocols approved by the Institutional Animal Care and Use Committee of the Seoul Yonsei Pharmaceutical University Experimental Animal Center.

### Statistical analysis

Statistical analysis was evaluated by one-way or two-way ANOVA using GraphPad Prism version 8.0 (GraphPad Software, CA, United States). Data are presented as the mean ± standard deviation. Statistical significance was indicated (*p < 0.05; **p < 0.01).

## Results

### Ivermectin exerts synergistic effects with gemcitabine and inhibits pancreatic cancer growth

**FIGURE 1:**
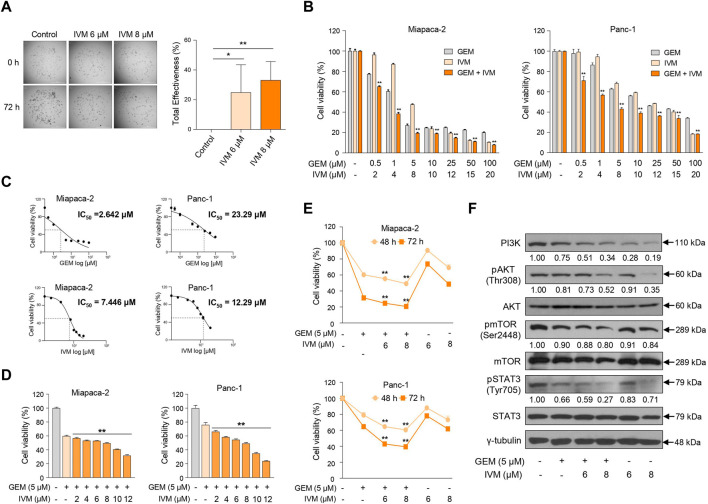
Ivermectin inhibits the proliferation of pancreas cancer cells. **(A)** Morphological changes in patient-derived organoids were monitored for 72 h after treatment with ivermectin at 6 and 8 μM concentrations. Total inhibiting effectiveness of ivermectin was calculated. Data represents the mean ± standard deviation (SD) (n = 5). *p < 0.05; **p < 0.01 compared with the control group. Scale bar = 1000 μm. **(B)** MIA PaCa-2 and PANC-1 cells were seeded in a 96-well plate and treated with increasing doses of gemcitabine and ivermectin for 72 h. Cell viability was determined using the water-soluble tetrazolium (WST) assay. **(C)** Median inhibitory concentration (IC_50_) values of gemcitabine and ivermectin for each cell line are shown. **(D)** MIA PaCa-2 and PANC-1 cells were treated with 5 μM gemcitabine and increasing doses of ivermectin for 48 h **(E)** MIA PaCa-2 and PANC-1 cells were treated with the indicated concentrations of gemcitabine and ivermectin for 48 and 72 h **(F)** MIA PaCa-2 cells were treated with gemcitabine and ivermectin at the indicated concentrations, and the protein expression levels were determined using western blotting. **(B,D,E)** Data represents the mean ± SD (*n* = 3). *p < 0.05 and **p < 0.01 compared with the gemcitabine alone group.

Ivermectin exerts antitumor effects in various cancer types (Juarez et al., 2018). However, the mechanism underlying its antitumor effect on pancreatic cancer remains unclear. To elucidate the effects of ivermectin and the underlying molecular mechanisms, we first tested the effect of ivermectin on pancreatic cancer proliferation using patient-derived organoids. Ivermectin significantly inhibited the growth of organoids in a concentration-dependent manner compared to the control group (Figure 1A), indicating that ivermectin inhibits the growth of pancreatic cancer.

Next, to determine whether the gemcitabine and ivermectin combination affected pancreatic cancer cell proliferation, pancreatic cancer cells were treated with either gemcitabine or ivermectin, and the viability of PANC-1 and MIA PaCa-2 cells were determined using the WST-1 assay. Ivermectin–gemcitabine combination significantly reduced the cell viability compared to gemcitabine alone (Figure 1B). Also, the median inhibitory concentration (IC_50_) value of each drug was calculated *via* IC_50_ analysis to decide the optimal concentrations of both drugs to treat pancreatic cancer (Figure 1C). The treatment concentration of gemcitabine for pancreatic cancer was determined as 5 μM, and gemcitabine was co-administered with various concentrations of ivermectin to pancreatic cancer cells. The cell viability decreased in a dose-dependent manner after 48 h (Figure 1D). We confirmed that the co-administration of ivermectin and gemcitabine significantly inhibited the cell proliferation in a concentration- and time-dependent manner (Figure 1E). We also examined cell proliferation-related genes following treatment with the indicated concentrations of gemcitabine and ivermectin (Figure 1F). The expression levels of cell proliferation-related genes were significantly reduced in the ivermectin–gemcitabine group than in the gemcitabine alone group. These results suggest that ivermectin significantly enhances the anti-proliferative effects of gemcitabine on cell growth.

### Ivermectin–gemcitabine combination induces cell cycle arrest in pancreatic cancer

**FIGURE 2:**
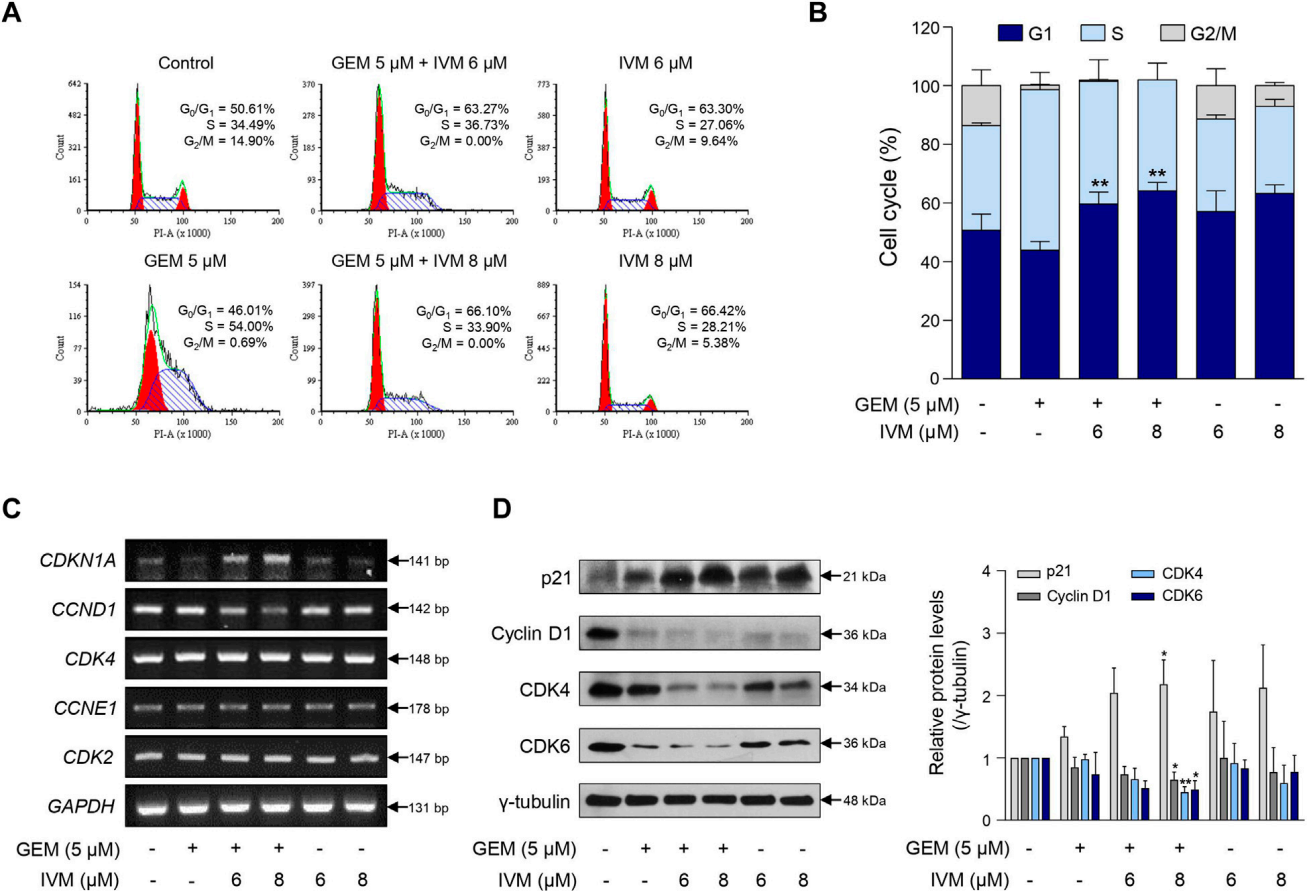
Ivermectin–gemcitabine combination induces G1 phase arrest. **(A)** MIA PaCa-2 cells were treated with the indicated concentrations of gemcitabine and ivermectin for 48 h and monitored by propidium iodide (PI) staining and flow cytometry. **(B)** Graph showing the percentage of cells at each stage of the cell cycle. Data represents the mean ± SD (*n* = 3). *p < 0.05 and **p < 0.01 compared with the gemcitabine alone group. **(C)** After the treatment of MIA PaCa-2 cells with gemcitabine and ivermectin, the mRNA expression levels of p21, cyclin D1, cyclin-dependent kinase (CDK)-4, cyclin E1, and CDK2 were estimated *via* polymerase chain reaction (PCR). **(D)** Protein levels of p21, cyclin D1, CDK4, and CDK6 were determined *via* western blotting of gemcitabine and ivermectin-treated cells. Data represents the mean ± SD (*n* = 3). *p < 0.05 and **p < 0.01 compared with the gemcitabine alone group.

Gemcitabine is a DNA-damaging drug that induces S/G2 phase arrest in bladder cancer (Montano et al., 2017), while ivermectin induces G1/S phase arrest in cervical cancer (Zhang et al., 2019). As ivermectin and gemcitabine treatment decreased cell viability, we performed cell cycle analysis using flow cytometry to confirm whether ivermectin and gemcitabine induced cell cycle arrest in pancreatic cancer. Gemcitabine induced S phase arrest, whereas ivermectin–gemcitabine combination treatment increased the percentage of G1 phase arrest cells in a dose-dependent manner (Figures 2A,B). These data suggest that the ivermectin–gemcitabine combination inhibits cell proliferation by inducing G1 arrest in pancreatic cancer cells.

Consistently, the ivermectin–gemcitabine combination affected the expression of cell cycle-related genes. Ivermectin–gemcitabine combination treatment increased p21 expression and decreased cyclin D1 expression more effectively than gemcitabine or ivermectin alone. However, there was no difference in the mRNA expression levels of cyclin-dependent kinase (CDK)-4, cyclin E1, and CDK2 (Figure 2C). Western blotting analysis was performed to confirm the regulation of genes at the protein level. The results showed that the combination treatment further reduced the expression levels of CDK4 and CDK6 compared to gemcitabine treatment alone (Figure 2D). These results indicate that the ivermectin–gemcitabine combination inhibits the formation of cyclin D1 and CDK4/6 complexes in pancreatic cancer, inhibits G1-S cell cycle transition, and induces G1 phase arrest.

### Ivermectin–gemcitabine combination enhances apoptosis more than gemcitabine alone

**FIGURE 3:**
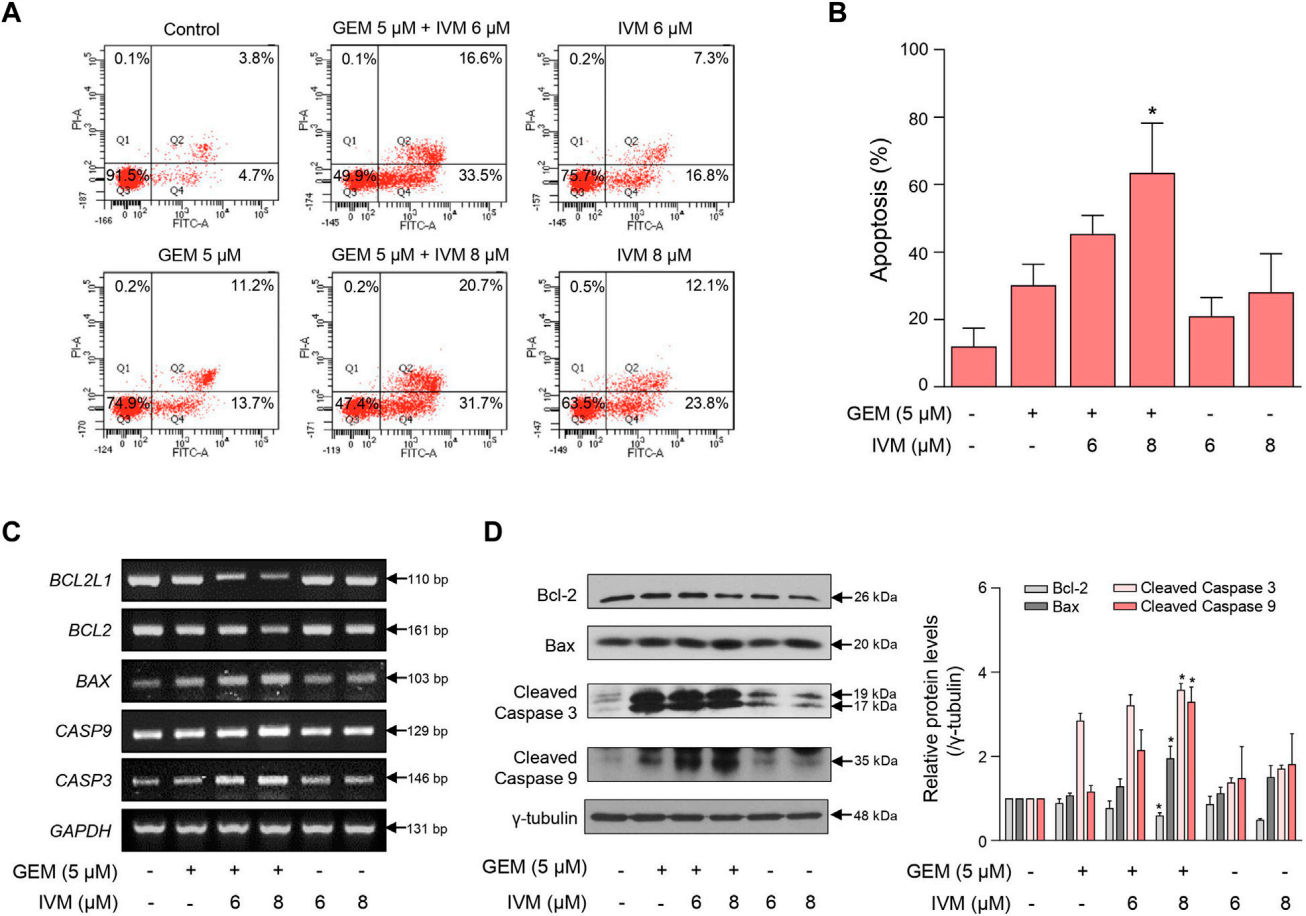
Ivermectin and gemcitabine combination increases cell apoptosis. **(A)** MIA PaCa-2 cells were treated with the indicated concentrations of gemcitabine and ivermectin for 48 h. Apoptosis of MIA PaCa-2 cells was analyzed using fluorescence-activated cell sorting (FACS) after Annexin V-fluorescein isothiocyanate (FITC) staining. **(B)** Graph showing the percentage of cells in early and late apoptosis Data represents the mean ± SD (*n* = 3). *p < 0.05 and **p < 0.01 compared with the gemcitabine alone group. **(C)** mRNA expression levels in cells treated with the ivermectin and gemcitabine combination for 48 h were determined using PCR. **(D)** Protein expression levels were determined using western blotting after ivermectin and gemcitabine treatment. Data represents the mean ± SD (*n* = 3). *p < 0.05 and **p < 0.01 compared with the gemcitabine alone group.

To investigate whether the ivermectin–gemcitabine combination promotes apoptosis, we performed fluorescence-activated cell sorting (FACS) analysis using Annexin V/PI dual staining after treatment with the indicated concentrations of ivermectin and gemcitabine. The ivermectin–gemcitabine combination showed a significantly higher rate of apoptosis than gemcitabine alone (Figures 3A,B). We then confirmed the expression of apoptosis-related genes at both the mRNA and protein levels. The ivermectin–gemcitabine combination significantly increased the expression levels of B-cell lymphoma-associated X, caspase 3, and caspase 9 and decreased the levels of B-cell lymphoma-extra-large and B-cell lymphoma-2 compared to gemcitabine alone (Figures 3C,D). These results suggest that the combination of ivermectin and gemcitabine synergistically increases apoptosis by regulating the proapoptotic factors in pancreatic cancer.

### Ivermectin–gemcitabine combination enhances apoptosis *via* mitochondrial dysfunction

**FIGURE 4:**
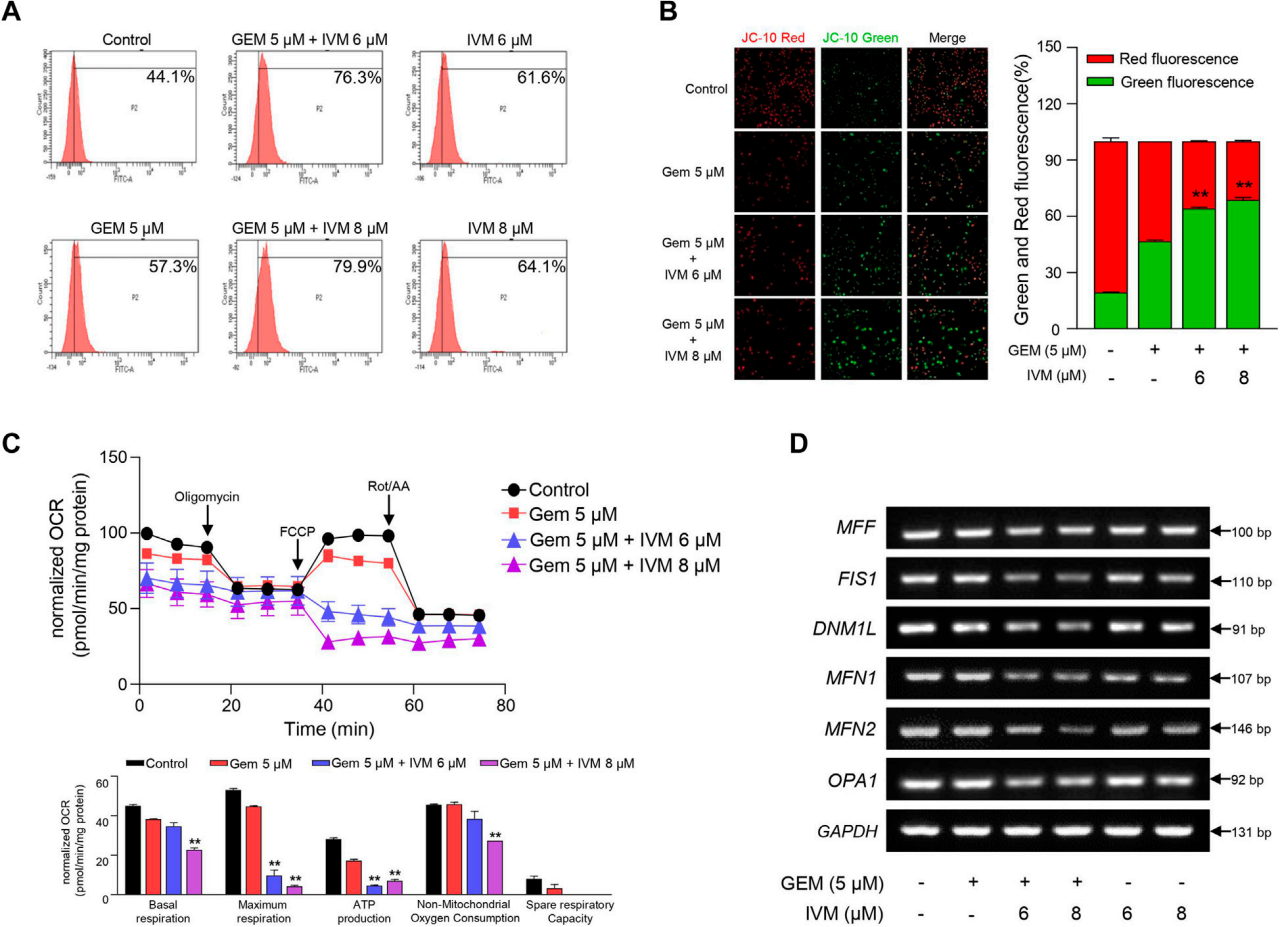
Combination of ivermectin and gemcitabine induces mitochondrial dysfunction *via* reactive oxygen species (ROS) generation. **(A)** Relative ROS production in MIA PaCa-2 cells treated with gemcitabine and ivermectin was analyzed using flow cytometry after dichlorofluorescein (DCF)-staining. **(B)** After JC-10 staining, mitochondrial membrane potential (MMP) was measured *via* fluorescence microscopy. Data represents the mean ± SD (*n* = 3). *p < 0.05 and **p < 0.01 compared with the gemcitabine alone group. **(C)** Oxygen consumption rate (OCR) was determined after treatment with ivermectin and gemcitabine and calculated under oligomycin, carbonyl cyanide p-(trifluoromethoxy) phenylhydrazone (FCCP), and Rot/antimycin A treatments. Data represents the mean ± SD (*n* = 3). *p < 0.05 and **p < 0.01 compared with the gemcitabine alone group. **(D)** mRNA expression levels of mitochondria fusion and fission genes were determined *via* PCR.

As various anticancer drugs have been reported to induce apoptosis due to overproduction of the oxidative stress cascade (Ding et al., 2016; Arfin et al., 2021), we measured cellular ROS production in ivermectin–gemcitabine combination-treated cells to confirm the status of oxidative stress. As expected, ivermectin–gemcitabine increased the fluorescence intensity of DCF-DA, indicating increased ROS generation (Figure 4A).

A high level of ROS increases membrane permeability and induces disruption of MMP (Li et al., 2013). MMP, a consequence of the electrochemical proton gradient maintained for ATP synthesis, is an important indicator of functional mitochondria (Perry et al., 2011). Therefore, we monitored the MMP levels in pancreatic cancer cells treated with ivermectin and gemcitabine. In normal cells, red fluorescence was detected by the JC-10 aggregates, whereas in apoptotic cells, green fluorescence was detected by the JC-10 monomer. The co-treatment group showed increased green fluorescence compared to gemcitabine alone (Figure 4B), suggesting that the ivermectin–gemcitabine combination treatment induces mitochondrial dysfunction with decreased levels of MMP due to increased levels of ROS.

As ROS impair the mitochondrial respiratory chain (Wang et al., 2015), we confirmed whether ROS produced by ivermectin and gemcitabine reduced OCR. As expected, ivermectin–gemcitabine co-administration significantly reduced mitochondrial respiration compared with gemcitabine alone (Figure 4C). Taken together, we found that the ivermectin–gemcitabine combination significantly induced apoptosis by activating pro-apoptotic factors through mitochondrial dysfunction caused by excessive ROS production compared to gemcitabine alone in pancreatic cancer.

### Ivermectin–gemcitabine combination inhibits mitophagy

During cancer progression, mitophagy can be more easily detected in cancer cells than in normal cells to manage the elevated ROS levels that cause apoptosis (Chiu et al., 2019; Wang Y. et al., 2020). Damaged mitochondria are usually detected as targets of mitophagy, which promotes mitochondrial fission (Wu et al., 2019). To determine whether ivermectin and gemcitabine affected mitophagy activation in pancreatic cancer, we investigated the expression of mitochondrial fusion- and fission-related genes. We found that the ivermectin–gemcitabine combination significantly reduced mitochondrial fusion- and fission-related gene expression compared with gemcitabine alone (Figure 4D). These data suggest that the two chemical compounds synergistically inhibited mitophagy by decreasing the expression of mitochondrial fission-related genes to induce apoptosis.

### Ivermectin–gemcitabine combination effectively suppresses the tumor growth

**FIGURE 5:**
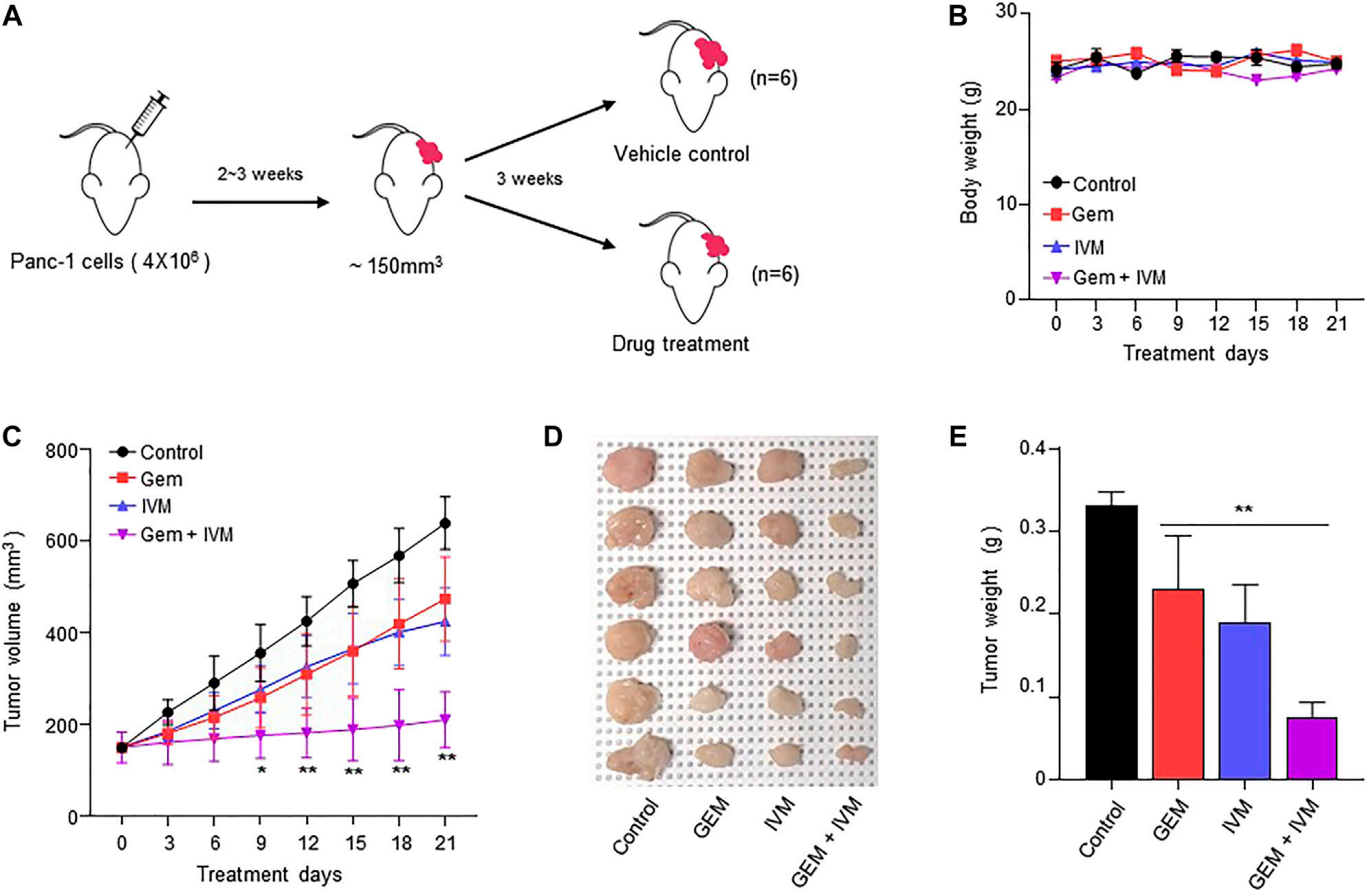
Combination of ivermectin and gemcitabine synergistically inhibits the tumor growth *in vivo*. **(A)** BALB/c nude mice were xenografted with PANC-1 cells (4 × 10^6^ cells). Then, the mice were intraperitoneally injected with dimethyl sulfoxide (DMSO) or gemcitabine (10 mg/kg) or ivermectin (5 mg/kg) twice a week for 21 days. **(B)** Mice body weights. **(C)** Tumor volume was measured every 3 days. Data represents the mean ± SD (*n* = 6). *p < 0.05 and **p < 0.01 compared with the gemcitabine alone group. **(D)** Representative image of tumors on day 21. **(E)** Tumor weights. Data represents the mean ± SD (*n* = 6). *p < 0.05 and **p < 0.01 compared with the gemcitabine alone group.

To evaluate the anti-proliferative effect of ivermectin–gemcitabine combination treatment *in vivo*, PANC-1 cells were injected subcutaneously into BALB/c nude mice and allowed to reach 150 mm^3^. Mice were randomly divided into four groups and ivermectin and/or gemcitabine were administered intraperitoneally twice a week (Figure 5A). There were no significant differences in body weight (Figure 5B). The combination of ivermectin and gemcitabine significantly suppressed tumor growth compared to the treatment with gemcitabine alone (Figure 5C). Both tumor size and weight were lower in the co-treatment group than in the gemcitabine alone group (Figures 5D,E). These results indicate that the ivermectin–gemcitabine combination has a synergistic effect in inhibiting the growth of pancreatic cancer.

## Discussion

Although gemcitabine is the first-line anticancer drug for pancreatic cancer, it does not significantly improve the survival rate of patients with pancreatic cancer. Therefore, gemcitabine-based combination therapies are being investigated to improve the treatment of patients with pancreatic cancer. Only a few known drugs can be used in combination with gemcitabine (Ramaswamy et al., 2017). Drug repurposing, a strategy that utilizes a drug that is already approved by the FDA by switching its original purpose to a new one, is also being actively studied as it is more cost-effective than creating new anticancer drugs (Zhang et al., 2020). Ivermectin, an antiparasitic drug, is being repurposed as an anticancer drug, and has been shown to synergize with doxycycline or tamoxifen in breast and prostate cancer (Juarez et al., 2020; Pfab et al., 2021). However, the exact role of ivermectin in pancreatic cancer has not yet been elucidated. In addition, the combined effects of ivermectin and gemcitabine have not yet been studied. In this study, we demonstrated that ivermectin can be used as an antitumor agent for pancreatic cancer. Moreover, combination treatment with gemcitabine suppressed the growth of cancer cells more effectively than gemcitabine alone.

Ivermectin inhibits cell proliferation *via* Akt/mTOR phosphorylation and induces G1 arrest in glioblastoma and cervical cancer (Liu et al., 2016; Juarez et al., 2018; Zhang et al., 2019). It synergistically increases the antitumor effects in colorectal cancer with vincristine, an anticancer agent, compared to ivermectin or vincristine alone (Jiang et al., 2019). Cell viability tests and FACS analysis suggested that ivermectin has an anti-proliferative effect and inhibits the cell cycle in pancreatic cancer. We investigated whether ivermectin synergistically enhances the anticancer effects of gemcitabine. We found that the combination treatment of ivermectin and gemcitabine significantly enhanced the antitumor effects *via* the phosphatidylinositol 3-kinase/mTOR/STAT3 pathway compared to gemcitabine treatment alone (Figure 1F).

Oxidative stress plays a predominant role in various cancers (Arfin et al., 2021) as the excessive accumulation of ROS can induce mitochondrial dysfunction and apoptosis (Garrido et al., 2006; Zhang et al., 2016). Ivermectin promotes programmed cell death *via* ROS production (Tang et al., 2021; Zhou et al., 2021), and gemcitabine induces DNA damage *via* ROS (Wang X. et al., 2020). In renal cancer, ivermectin promotes programmed cell death *via* mitochondrial dysfunction caused by ROS generation (Zhu et al., 2017). Ivermectin increases cell apoptosis (Figure 3); however, this function has not yet been elucidated in pancreatic cancer. Thus, in this study, oxidative stress induced by ivermectin and/or gemcitabine was confirmed by ROS production. To the best of our knowledge, this is the first report to demonstrate the function of ivermectin in pancreatic cancer. Ivermectin–gemcitabine combination significantly increased the ROS levels compared to gemcitabine alone (Figure 4A). This result indicates that oxidative stress contributes to pancreatic cancer apoptosis, suggesting that ivermectin may represent a therapeutic alternative for pancreatic cancer. As ROS reduce MMP and OCR by damaging mitochondria (Yang et al., 2021), the MMP and OCR values in the combination treatment group and gemcitabine alone group were compared. The ivermectin–gemcitabine combination further decreased MMP and OCR compared to gemcitabine alone (Figures 4B,C), indicating that the combination treatment promotes apoptosis in pancreatic cancer *via* mitochondrial dysfunction caused by ROS generation.

Mitochondrial biosynthesis is important for maintaining mitochondrial homeostasis, which is crucial for cell survival (Ma et al., 2020). Cancer induces mitochondrial fission and mitophagy to eliminate dysfunctional mitochondria, which can lead to cell death (Martinez-Outschoorn et al., 2011; Chourasia et al., 2015). Ivermectin–gemcitabine combination treatment significantly reduced the expression levels of the mitochondrial fission- and fusion-related genes compared to gemcitabine alone (Figure 4D). These results suggest that co-treatment with ivermectin and gemcitabine can inhibit the survival rate of cancer cells by blocking mitophagy.

Overall, our study showed that the combination of ivermectin and gemcitabine has a stronger antitumor effect on pancreatic cancer than gemcitabine alone. The ivermectin–gemcitabine combination increased apoptosis of pancreatic cancer cells *via* ROS-induced mitochondrial dysfunction. Moreover, the combination treatment reduced mitophagy, leading to cancer cell death, and further inhibited tumor growth *in vivo*. Therefore, the ivermectin–gemcitabine combination may be a promising therapeutic agent for improving the survival rate of patients with pancreatic cancer.

## Data Availability

The original contributions presented in the study are included in the article/Supplementary Material, further inquiries can be directed to the corresponding author.
