# Supplementary figures and images for: Ivermectin and gemcitabine combination treatment induces apoptosis of pancreatic cancer cells via mitochondrial dysfunction
Source: Front Pharmacol. 2022 Aug 26;13:934746. doi: 10.3389/fphar.2022.934746 (PMC9459089; doi:10.3389/fphar.2022.934746)

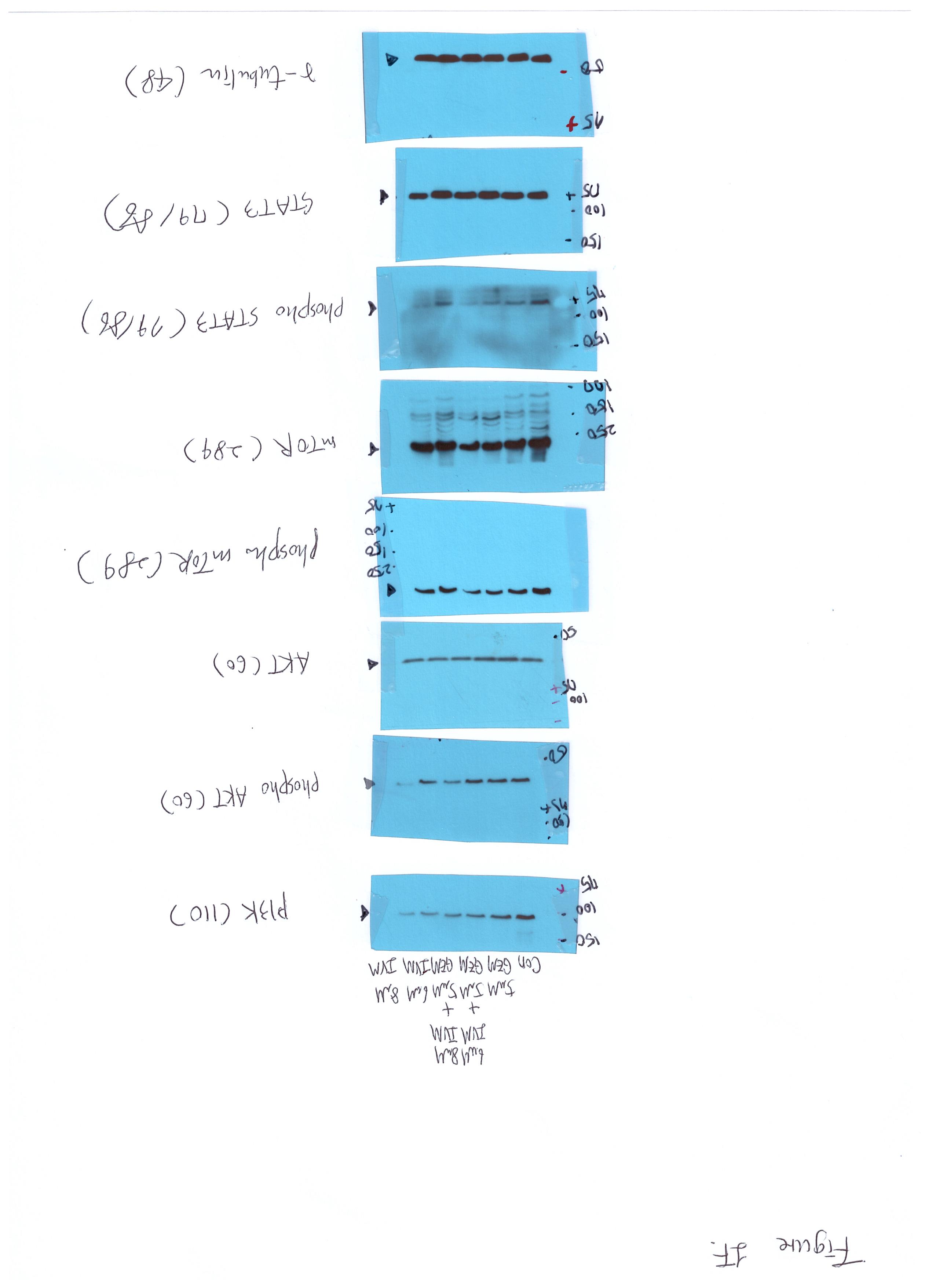

Supplement: Supplementary file 1 [file DataSheet1.ZIP › Fig 1/Figure 1F.jpg]

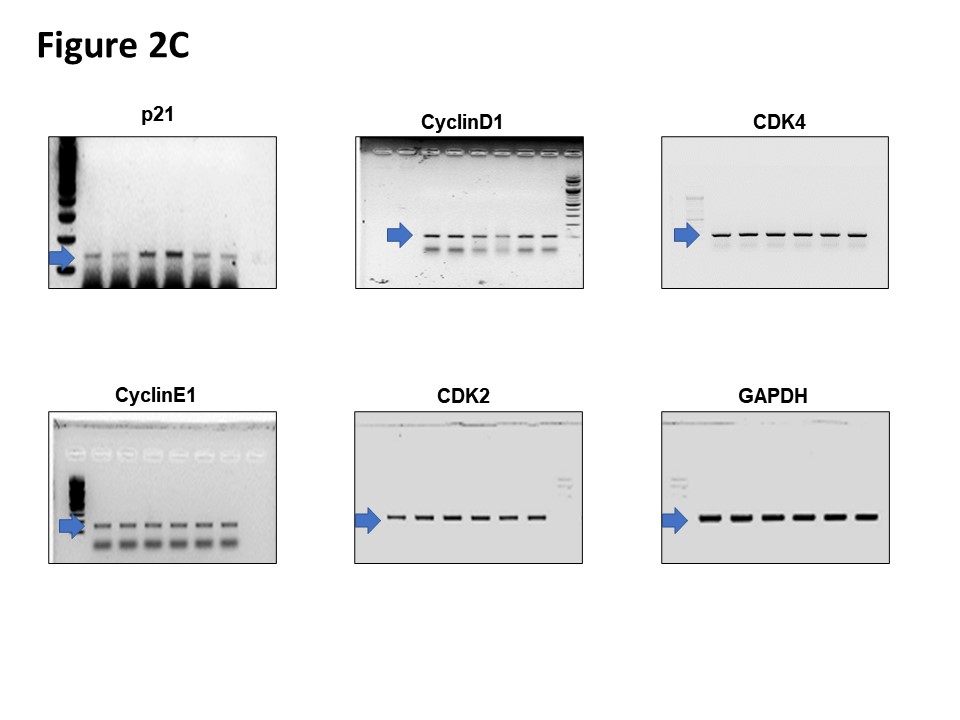

Supplement: Supplementary file 1 [file DataSheet1.ZIP › Fig 2/Fig 2C.JPG]

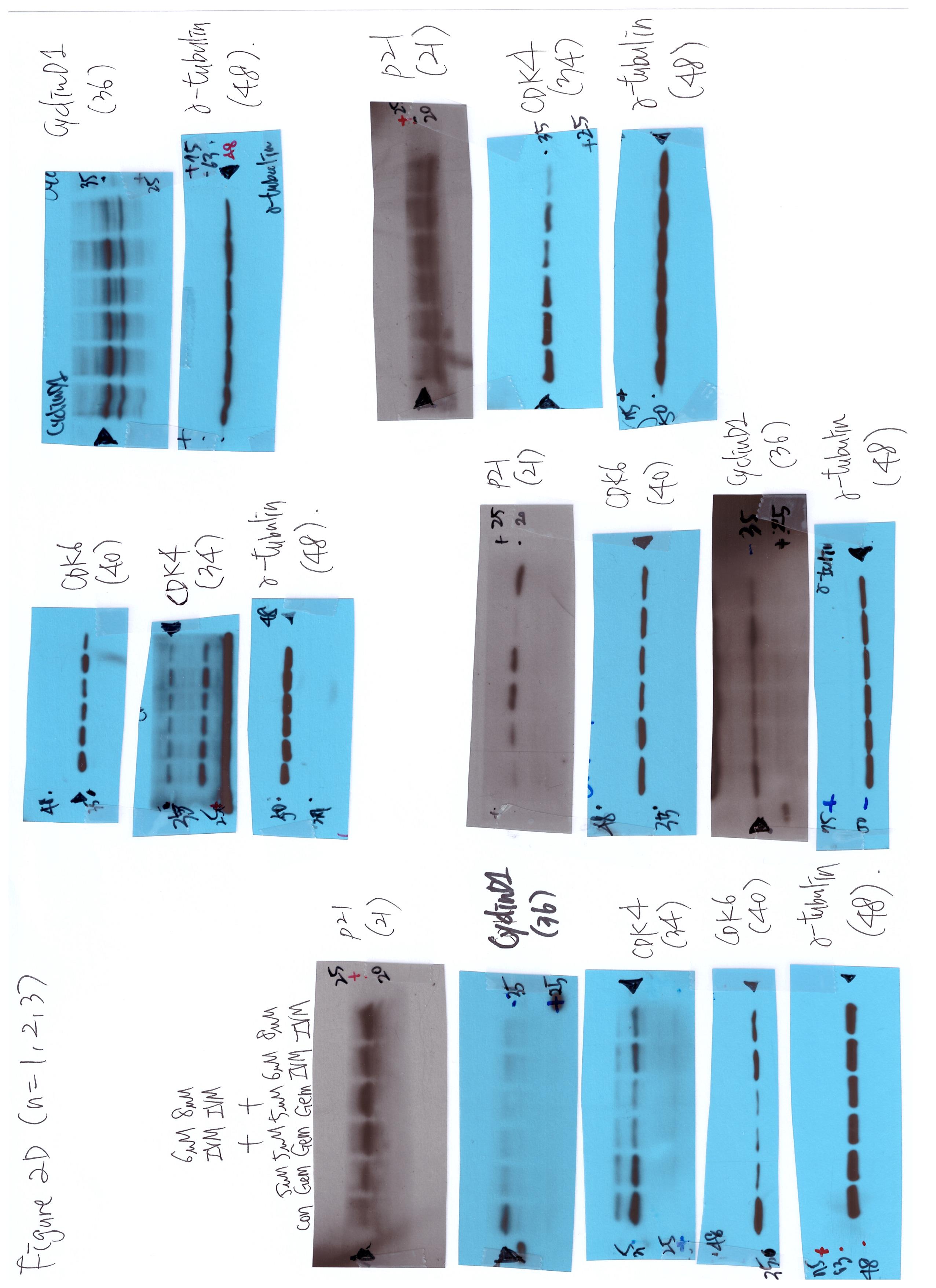

Supplement: Supplementary file 1 [file DataSheet1.ZIP › Fig 2/Fig 2D.jpg]

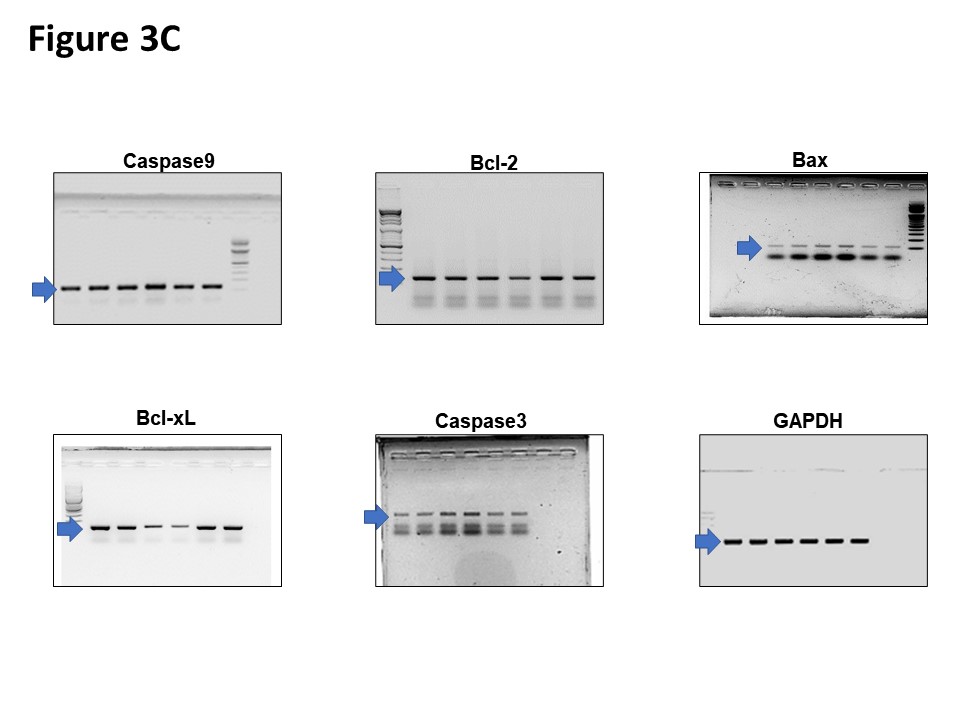

Supplement: Supplementary file 1 [file DataSheet1.ZIP › Fig 3/Fig 3C.JPG]

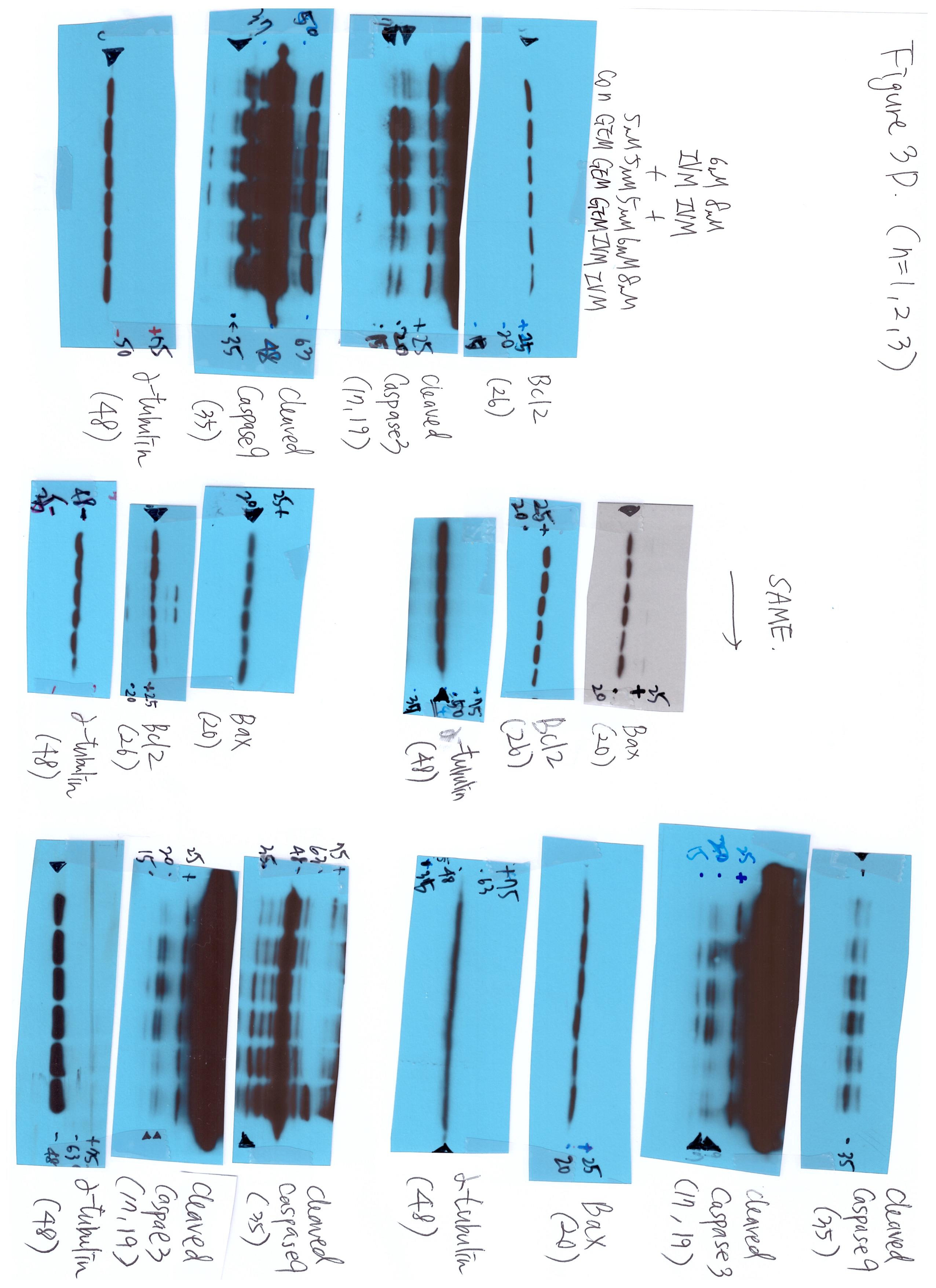

Supplement: Supplementary file 1 [file DataSheet1.ZIP › Fig 3/Fig 3D.jpg]

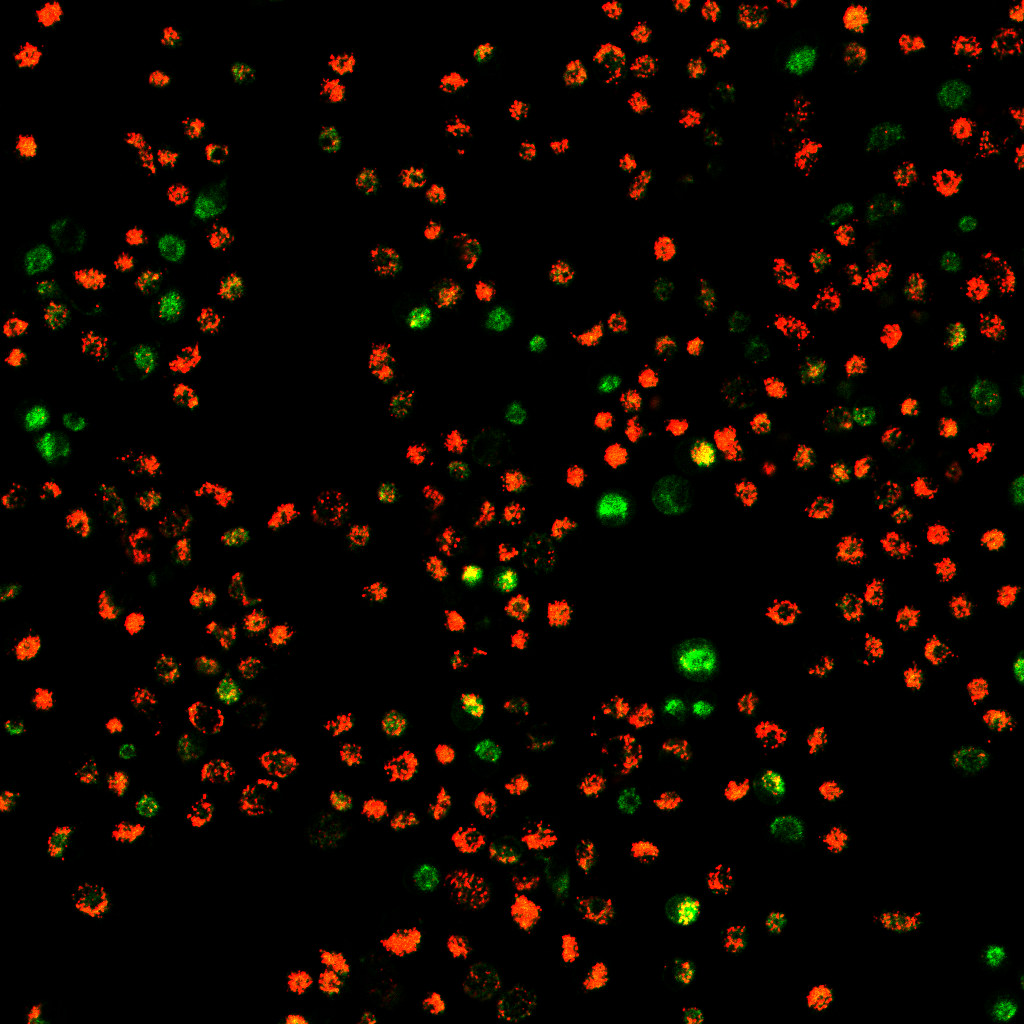

Supplement: Supplementary file 1 [file DataSheet1.ZIP › Fig 4/Fig 4B/Fig 4B con n=1.jpg]

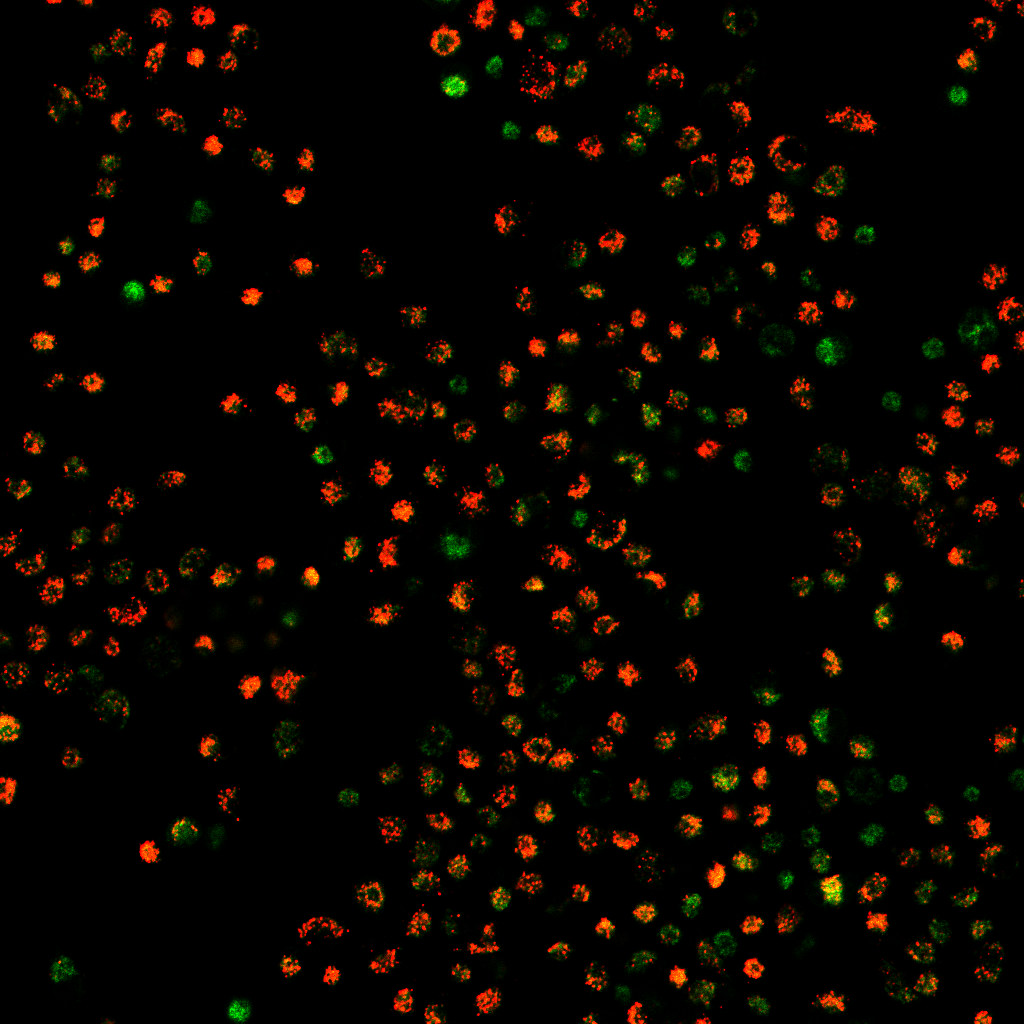

Supplement: Supplementary file 1 [file DataSheet1.ZIP › Fig 4/Fig 4B/Fig 4B con n=2.jpg]

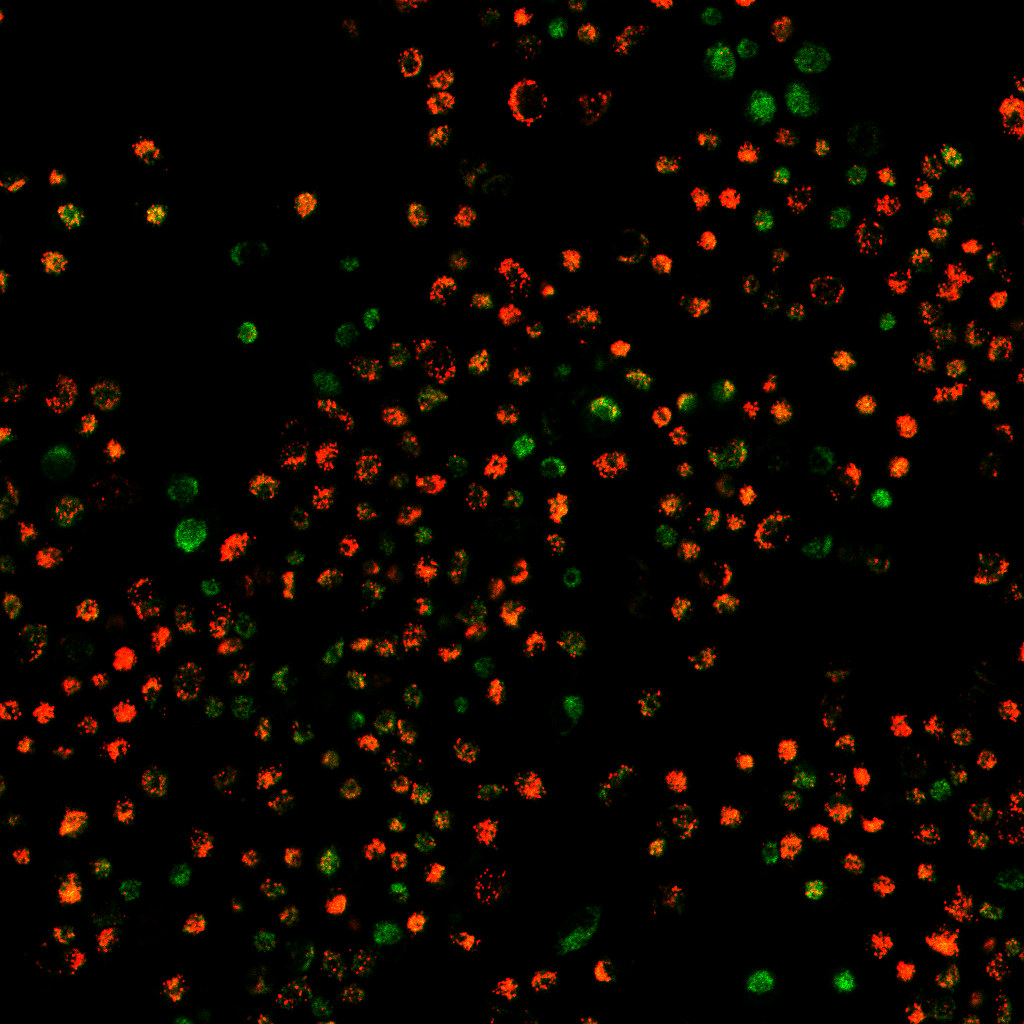

Supplement: Supplementary file 1 [file DataSheet1.ZIP › Fig 4/Fig 4B/Fig 4B con n=3.jpg]

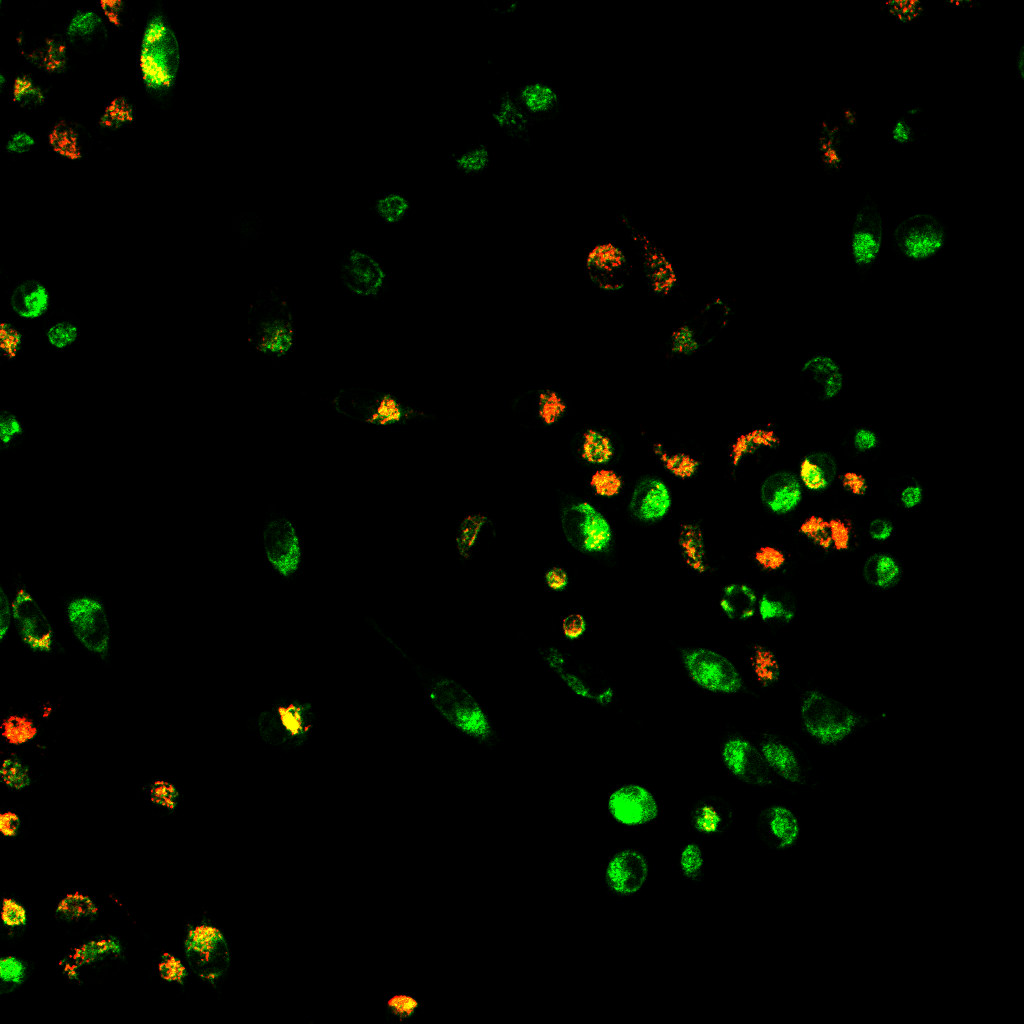

Supplement: Supplementary file 1 [file DataSheet1.ZIP › Fig 4/Fig 4B/Fig 4B gem 5uM ivm 6uM n=1.jpg]

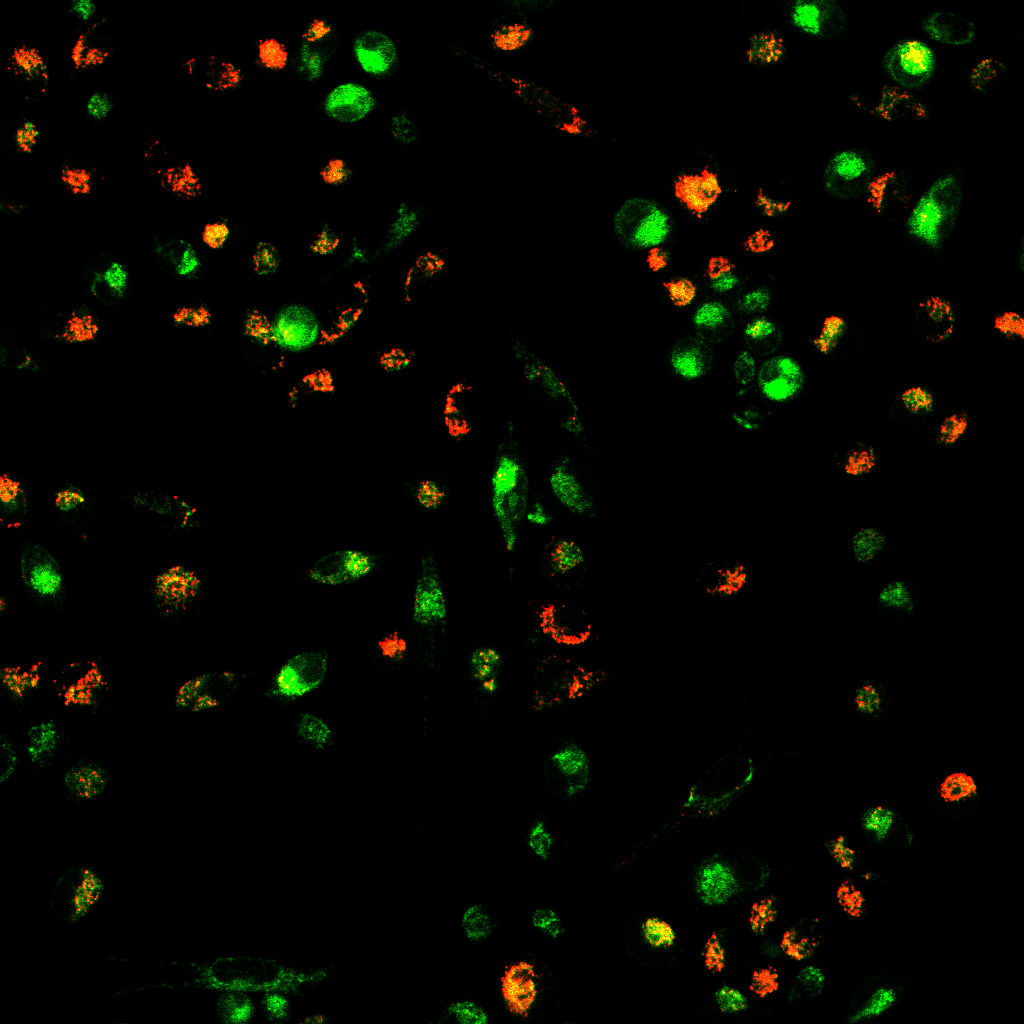

Supplement: Supplementary file 1 [file DataSheet1.ZIP › Fig 4/Fig 4B/Fig 4B gem 5uM ivm 6uM n=2.jpg]

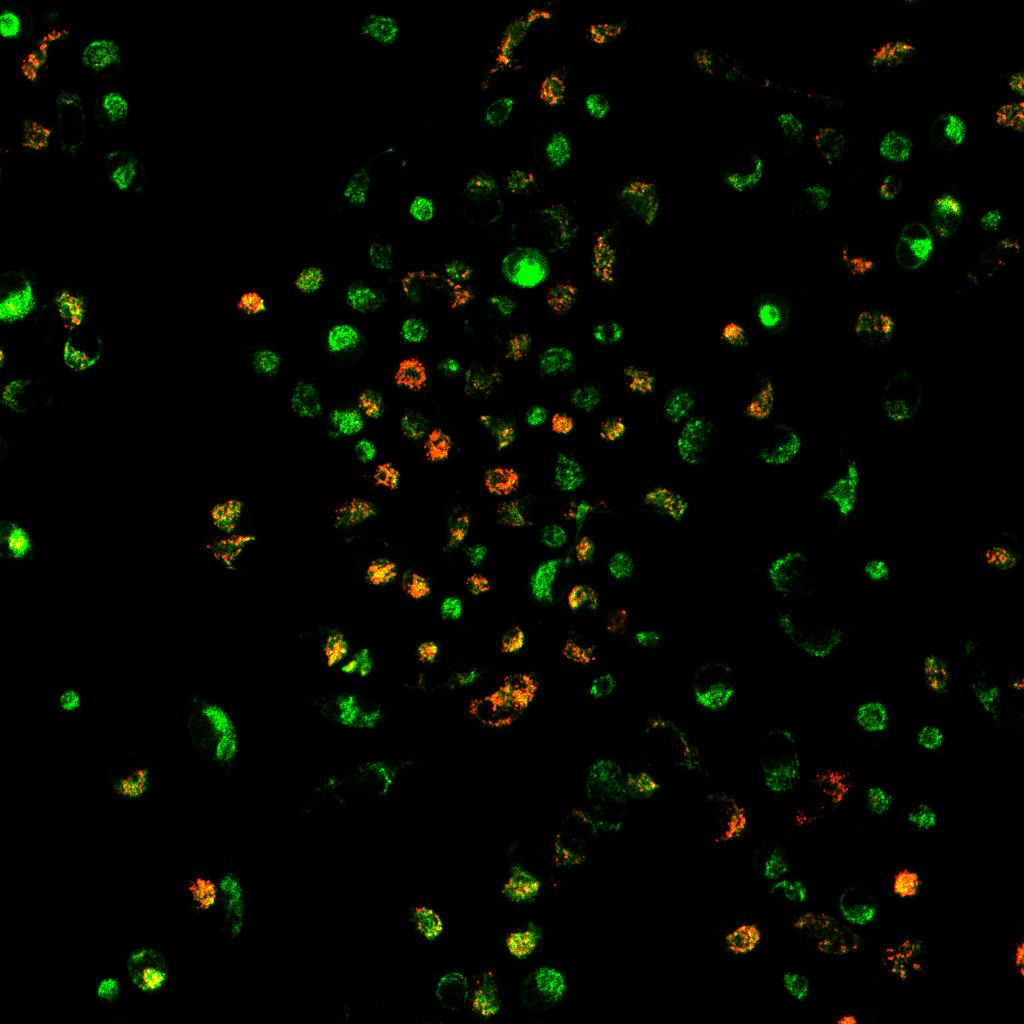

Supplement: Supplementary file 1 [file DataSheet1.ZIP › Fig 4/Fig 4B/Fig 4B gem 5uM ivm 6uM n=3.jpg]

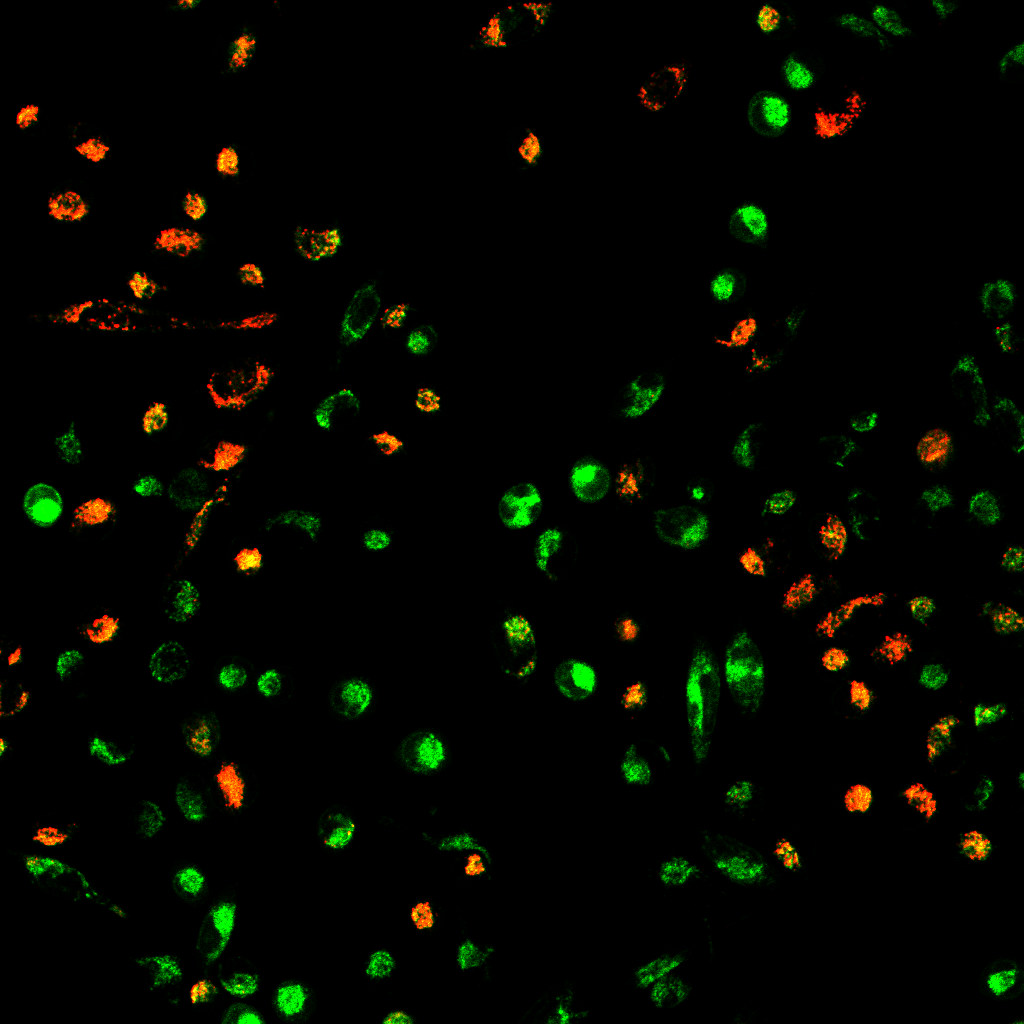

Supplement: Supplementary file 1 [file DataSheet1.ZIP › Fig 4/Fig 4B/Fig 4B gem 5uM ivm 8uM n=1.jpg]

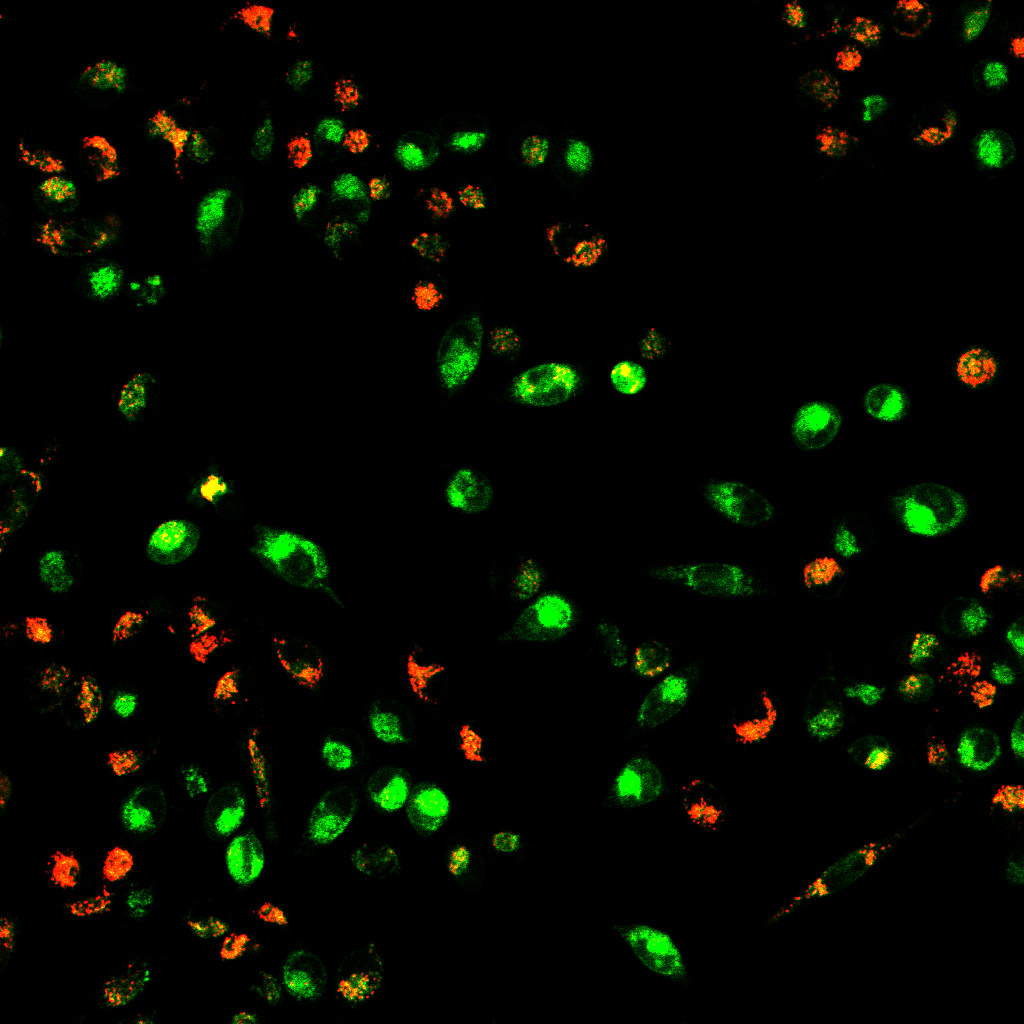

Supplement: Supplementary file 1 [file DataSheet1.ZIP › Fig 4/Fig 4B/Fig 4B gem 5uM ivm 8uM n=2.jpg]

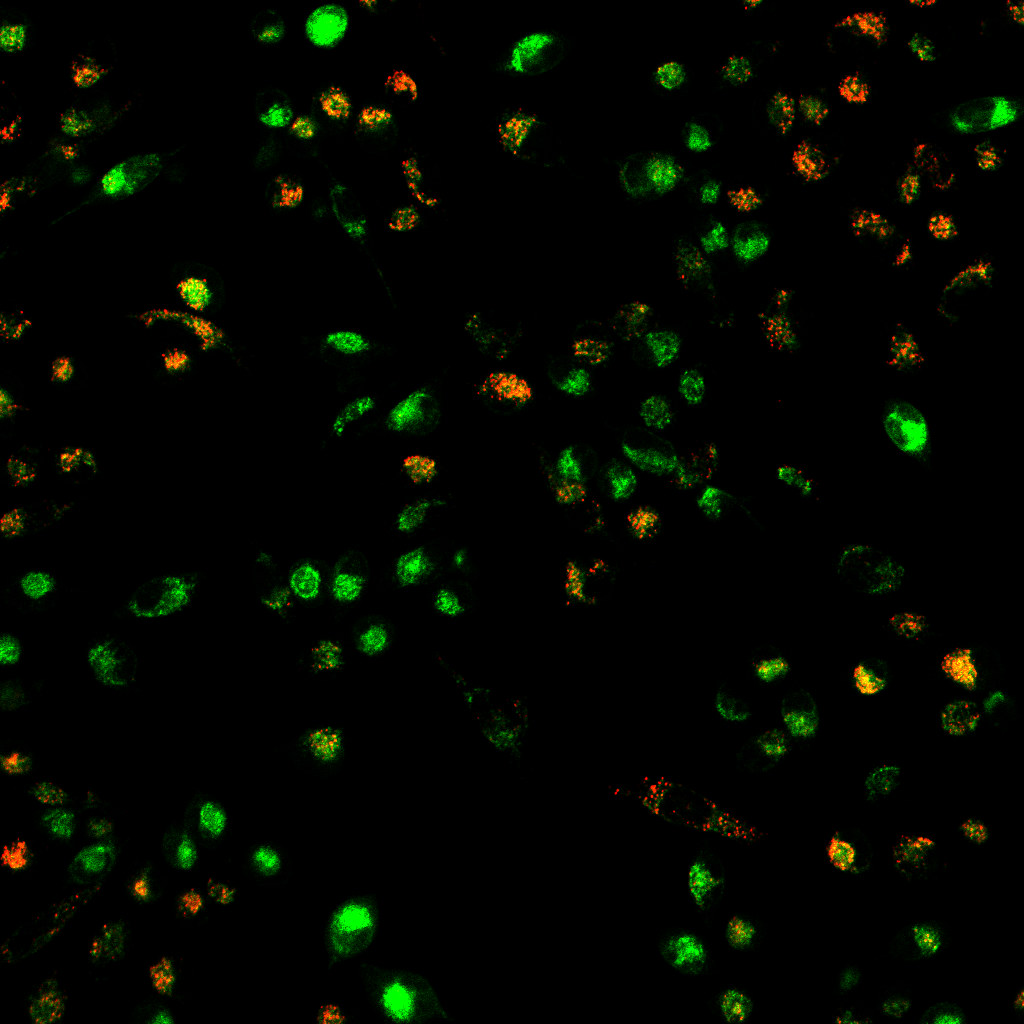

Supplement: Supplementary file 1 [file DataSheet1.ZIP › Fig 4/Fig 4B/Fig 4B gem 5uM ivm 8uM n=3.jpg]

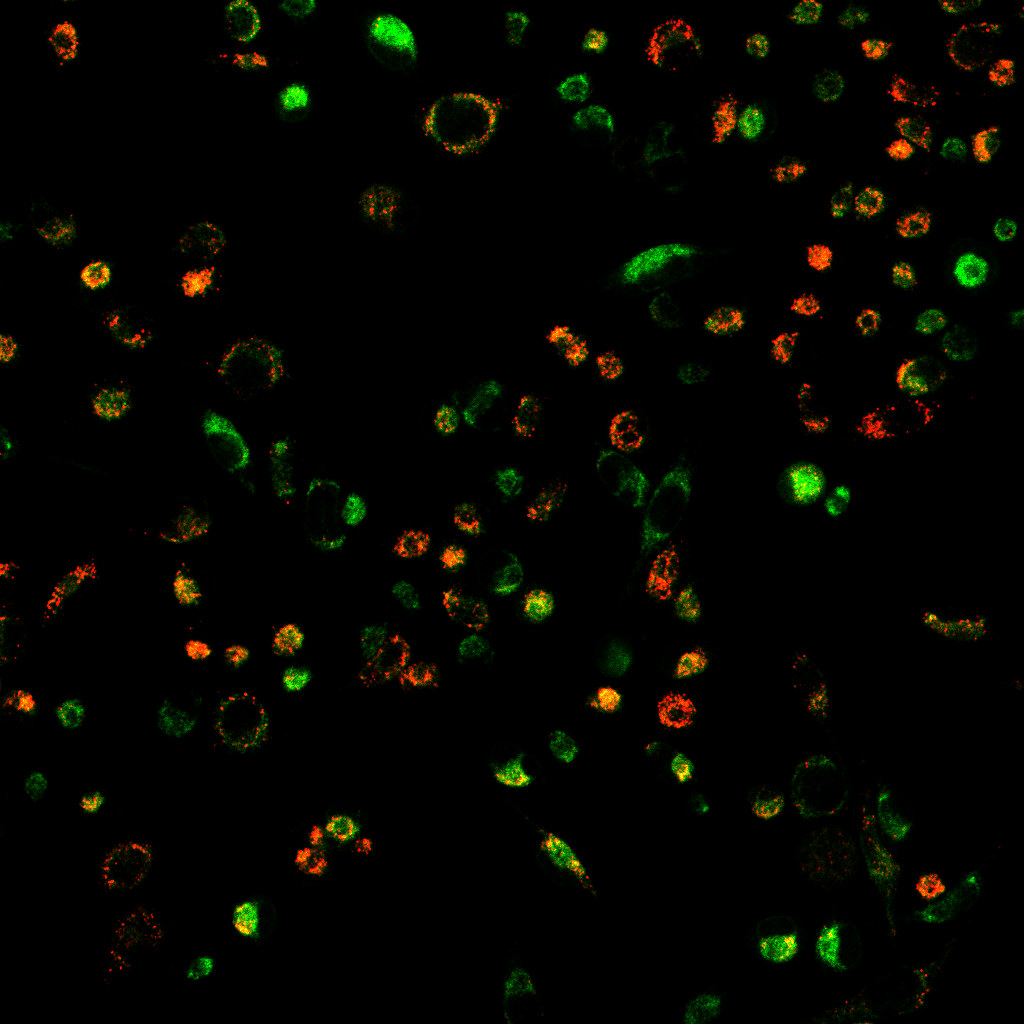

Supplement: Supplementary file 1 [file DataSheet1.ZIP › Fig 4/Fig 4B/Fig 4B gem 5uM n=1.jpg]

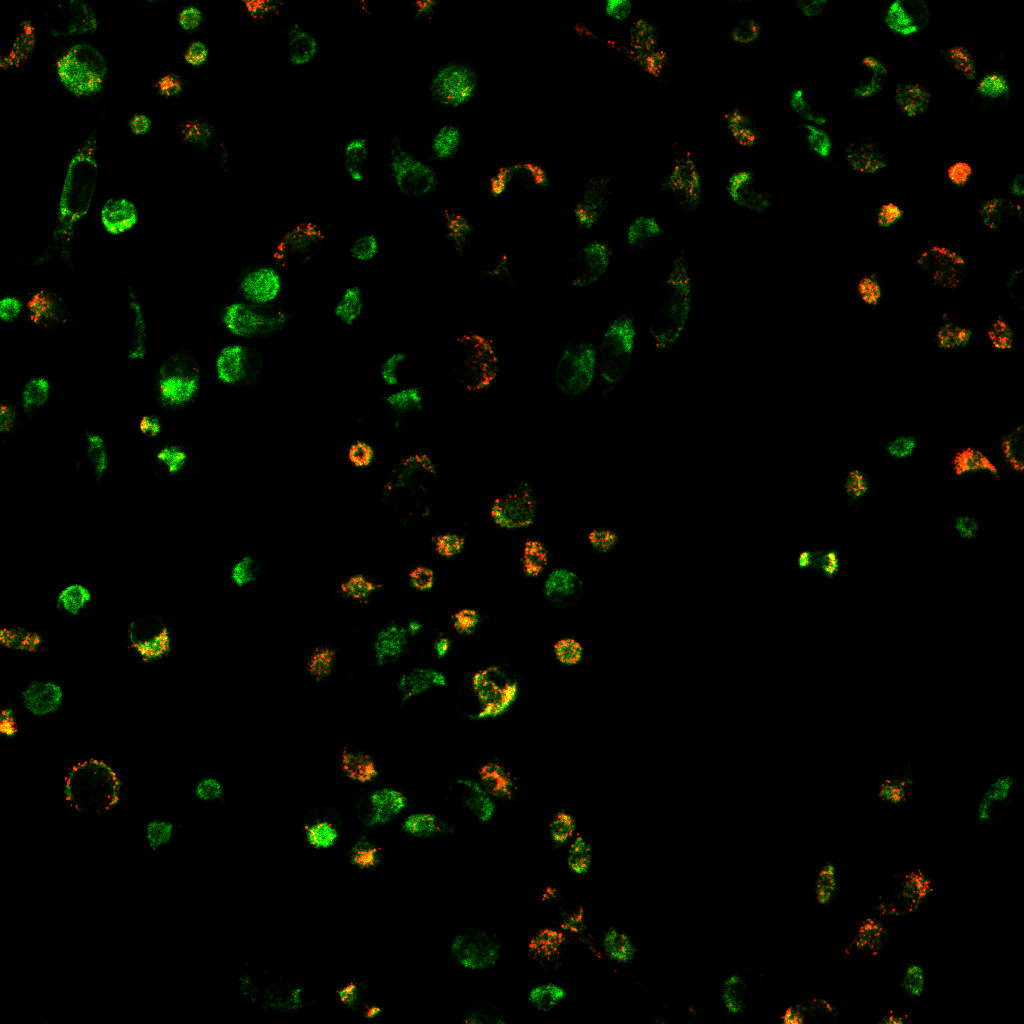

Supplement: Supplementary file 1 [file DataSheet1.ZIP › Fig 4/Fig 4B/Fig 4B gem 5uM n=2.jpg]

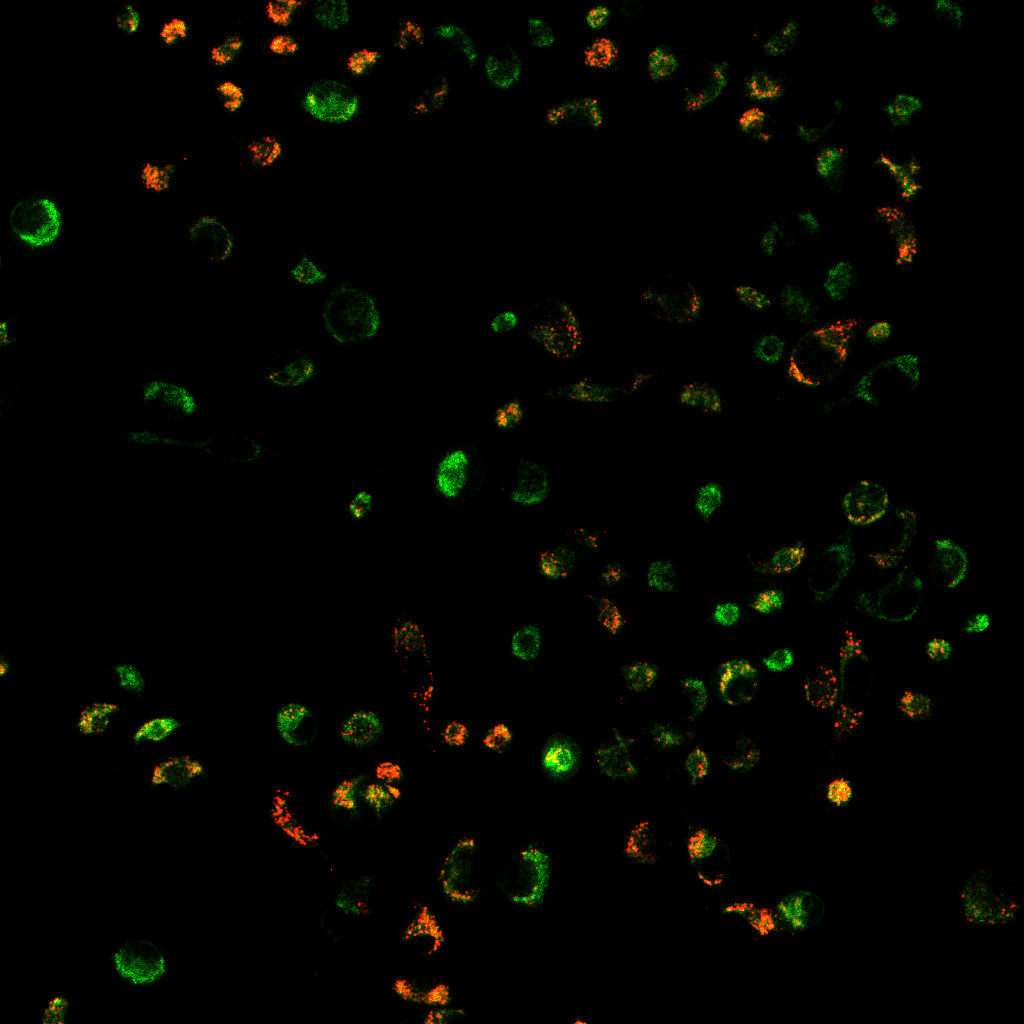

Supplement: Supplementary file 1 [file DataSheet1.ZIP › Fig 4/Fig 4B/Fig 4B gem 5uM n=3.jpg]

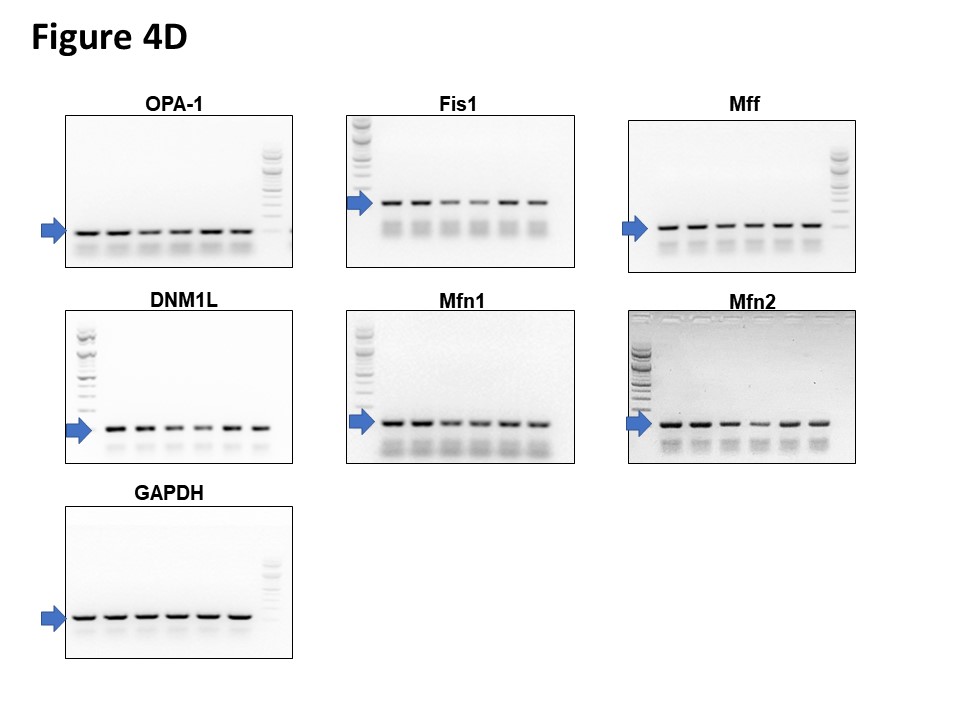

Supplement: Supplementary file 1 [file DataSheet1.ZIP › Fig 4/Fig 4D.JPG]
